# A systematic review of the intervention components, adherence and outcomes of enhanced recovery programmes in older patients undergoing elective colorectal surgery

**DOI:** 10.1186/s12877-019-1158-3

**Published:** 2019-06-06

**Authors:** Katleen Fagard, Albert Wolthuis, André D’Hoore, Marleen Verhaegen, Jos Tournoy, Johan Flamaing, Mieke Deschodt

**Affiliations:** 1 Department of Geriatric Medicine, University Hospitals Leuven, Leuven, Belgium; 2 Department of Abdominal Surgery, University Hospitals Leuven, Leuven, Belgium; 3 Department of Abdominal Surgical Oncology, KU Leuven, Leuven, Belgium; 4 Department of Anaesthesia, University Hospitals Leuven, Leuven, Belgium; 5 Department of Chronic Diseases, Metabolism and Ageing (CHROMETA), KU Leuven, Leuven, Belgium; 6 Institute of Nursing Science, Department of Public Health, University of Basel, Basel, Switzerland

**Keywords:** Aged, Aged, 80 and over, Colorectal surgery, Enhanced recovery, Fast track

## Abstract

**Background:**

Enhanced recovery programmes (ERPs) aim to attenuate the surgical stress response and accelerate recovery after surgery, but are not specifically designed for older patients. The objective of this study was to review the components, adherence and outcomes of ERPs in older patients (≥65 years) undergoing elective colorectal surgery.

**Methods:**

Pubmed, Embase and Cinahl were searched between 2000 and 2017 for randomised and non-randomised controlled trials, before-after studies, and observational studies. The methodological quality of the studies was evaluated using the MINORS quality assessment. The review was performed and reported according to the PRISMA guidelines.

**Results:**

Twenty-one studies, including 3495 ERP patients aged ≥65 years, were identified. The ERPs consisted of a median of 13 intervention components. Adherence rates were reported in 9 studies and were the highest (≥80%) for pre-admission counselling, no bowel preparation, limited pre-operative fasting, antithrombotic and antimicrobial prophylaxis, no nasogastric tube, active warming, and limited intra-operative fluids. The median post-operative length of stay was 6 days. The median post-operative morbidity rate (Clavien-Dindo I-IV) was 23.5% in-hospital and 29.8% at 30 days. The in-hospital post-operative mortality rate was 0% in most studies and amounted to a median of 1.4% at 30 days. The median 30-day readmission rate was 4.9% and the median reoperation rate was 5.0%.

**Conclusions:**

ERPs in older patients were in accordance with the ERP consensus guidelines. Although the number of intervention components applied increased over time, outcomes in earlier and later studies remained comparable. Adherence rates were under-reported. Future studies should explore adherence and age-related factors, such as frailty profile, that could influence adherence.

**Trial registration:**

PROSPERO 2018 CRD42018084756.

**Electronic supplementary material:**

The online version of this article (10.1186/s12877-019-1158-3) contains supplementary material, which is available to authorized users.

## Background

Fast Track protocols, also known as Enhanced Recovery After Surgery (ERAS®), or Enhanced Recovery Programmes (ERPs), have been developed by surgeons and anaesthesiologists to reduce the surgical stress response, accelerate recovery, and improve overall post-operative outcomes [1]. They were initially introduced in the early nineties by Kehlet and colleagues as standard of care for colorectal surgery, and have spread to other surgical specialties [1, 2]. ERPs generally include about 20 evidence-based intervention components during the peri-operative period, and require the active participation of a multidisciplinary team and the patient [3–5].

Due to demographic aging and advances in surgical and anaesthetic techniques, the demand for surgical procedures in older persons is rapidly increasing [6, 7], but the ability of older patients to actively participate in ERPs and to achieve the same results as younger patients has been debated [8, 9]. After all, ERPs were not specifically designed for older patients. On the other hand, older patients might actually benefit more than younger patients, because they are more susceptible to adverse post-operative outcomes and longer hospital stays [9, 10].

In 2014, Bagnall et al. published a first review about the safety, feasibility and efficacy of ERPs in patients aged 65 and over undergoing colorectal surgery, including 16 studies published before February 2014 [11]. Launay-Savary et al. performed a new search until 2015 and included two extra studies: a meeting abstract and a study later retracted from literature [12]. With new literature emerging since the latest reviews, and ERPs being further standardised, a new systematic literature review was deemed useful.

The primary objective of this review was to map intervention components of ERPs in patients aged 65 years and older undergoing elective colorectal surgery. In addition, we analysed adherence to individual ERP components and outcomes of the ERPs in patients aged 65 years and older.

## Methods

This review was performed in the framework of the PRISMA guidelines (www.prisma-statement.org) and was registered in PROSPERO, the international prospective register of systematic reviews (registration number CRD42018084756).

### Search strategy

An electronic bibliographic database search through PUBMED, EMBASE, and CINAHL was performed.

The following search string was used for PubMed and adapted for the other databases (Additional file 1): (((“colorectal surgery”[mesh]) OR ((colorectal[tiab] OR colon[tiab] OR colonic[tiab] OR colectomy[tiab] OR rectum[tiab] OR rectal[tiab] OR pelvic[tiab]) AND (surgery[tiab] OR surgical[tiab] OR operation[tiab] OR operative[tiab] OR resection[tiab]))) AND (ERAS[tiab] OR “enhanced recovery” [tiab] OR “accelerated recovery” [tiab] OR “expedited recovery” [tiab] OR “fast track” [tiab] OR multimodal[tiab] OR multi-modal[tiab])). The search was limited to English, Dutch, French, German and Spanish articles published between January 1, 2000 and November 17, 2017. Reference lists and PubMed-citations of the included articles, as well as former systematic review articles related to the topic, were cross-referenced to retrieve additional relevant studies.

### Selection of relevant papers

All studies including adults aged 65 and over, undergoing elective colorectal surgery, were eligible for inclusion. The individual components of the applied ERP had to be described in detail, including at least one of the following outcomes for the (subgroup of) older patients: length of stay (LOS) or post-operative morbidity. Study designs included were randomised and non-randomised controlled trials, before-after studies and prospective and retrospective observational studies. Studies were excluded if they included patients undergoing emergency surgery, if they referred to general guidelines instead of giving a detailed description of their ERP protocol, if they focused on limited (< 5) intervention components, or if no full text was available. Two reviewers (KF, MP) independently screened the titles and abstracts of the articles retrieved by the search and removed duplicate articles, using Endnote. Full texts of relevant abstracts and articles obtained by cross-referencing were assessed for inclusion (KF, MM). Any discrepancies were discussed with a fourth reviewer (MD).

### Data extraction and synthesis

Two reviewers (MP, MM) extracted data, each from half of the included studies, using standardised data extraction forms. A third reviewer (KF) double-checked the correctness and completeness of the extracted data. In case of disagreement, a fourth reviewer (MD) was consulted. The following characteristics of the included studies were extracted: first author, year of publication, country and setting, study design, study population, in- and exclusion criteria, sample and age distribution, and the subsample of older people in an ERP included in this review. The reported ERP intervention components were grouped into 20 key components, in line with the 2012 ERAS® Society guidelines for peri-operative care in elective colonic and rectal/pelvic surgery [3, 4]. The total number of studies including a certain ERP intervention component as well as the total number of ERP intervention components per study were calculated. Apart from LOS and post-operative morbidity, the following outcomes, if available, were reported: post-operative mortality, time to reach discharge criteria, adherence to ERP components, 30-day readmission rate and reoperation rate. Post-operative morbidity and mortality were considered in-hospital and within 30 days of surgery. Post-operative morbidity was reported as the percentage of patients experiencing post-operative complications (severity grades I to IV according to the Clavien-Dindo classification) [13]. If the study reported the percentage of older patients in which an intended ERP component was actually applied, this percentage was considered as the adherence to that ERP component. The most frequently reported discharge criteria, also referred to as ERP recovery goals, were summarised by reporting the time (in days or hours) to reach each recovery goal (i.e. no morbidity evidence, ambulation, first flatus, first stool, oral intake, pain control with oral drugs) [1, 14].

### Risk of bias (quality) assessment

The methodological quality of the included studies was assessed independently by three reviewers (KF, MP and MM) through the Methodological Index for Non-Randomised Studies (MINORS), containing eight items (the maximum item score per item is 2, the ideal global score 16) [15]. Any discrepancies were discussed and agreed upon with a fourth reviewer (MD).

### Data synthesis and analysis

Results from individual studies and study groups are provided in the tables as percentages and averages (means or medians). To summarize the data in the manuscript, medians and ranges across study groups were calculated. Due to many differences among studies regarding study population, age groups, ERPs, risk of bias, definitions of outcomes and other methodological aspects, a formal meta-analysis was not performed.

## Results

### Article selection

**Fig. 1:**
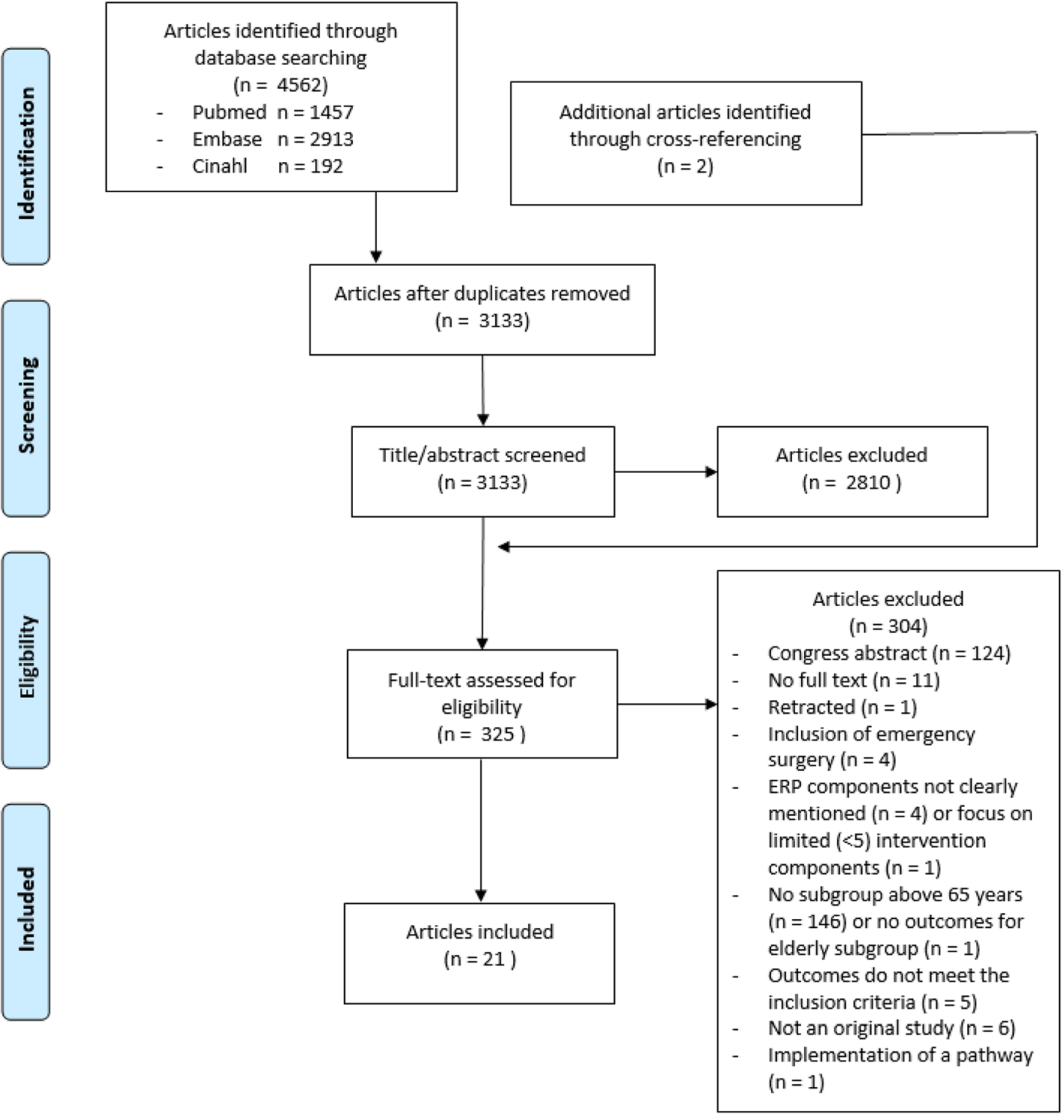
PRISMA flowchart showing the study selection process

The search generated 4562 articles (Fig. 1). After removing 1429 duplicates and excluding 2810 articles based on title and abstract, 323 full texts plus 2 additional articles found by cross-referencing, were evaluated for inclusion. Finally, 21 articles were included [16–36]. Five of the included articles did not provide ERP details, but were included because the applied ERP was described in detail in another publication [18, 21, 22, 25, 26, 33]. Although inclusion periods of the two studies published by Braga et al. partially overlapped, we reported the results as two individual studies [19, 20].

### Study and patient characteristics

Three studies compared ERPs with conventional care in an older age group [16, 24, 28] (Table 1). Ten studies compared ERPs in old versus young patient groups [17, 18, 20, 22, 23, 25, 27, 29, 31, 35], while 2 studies compared ERPs in different older age groups [19, 32]. Three studies described ERPs in a single older age group [21, 34, 36]. Three studies investigated patient factors that influence ERP outcomes, including age [26, 30, 33]. A total of 7610 patients were included in all studies, of whom 3495 (46%) patients (those ≥65 years in whom an ERP was applied) were considered in this review. Four studies divided the older patients in different age groups [17–19, 32]. As a consequence, the 21 studies included in this review comprised 26 study groups.

**Table 1:** Study and patient characteristics (original studies included in this review)

Original study	Country and setting	Study-design	Population	Sample	Age median (range) or mean (±SD), in y	Inclusion criteria (original study)	Exclusion criteria (original study)
Zeng 2017 [16]	China Monocentric, academic	Database analysis	Colorectal Laparoscopic Cancer	**94 ERP ≥ 75y** 157 CC ≥75y	**78 (75–88)** 78 (75–90)	≥75y Laparoscopic colorectal surgery Colorectal cancer	Emergency surgery Non-radical resection TNM stage IV Multi-organ resection
Pirrera 2017 [17]	Italy Monocentric, non-academic	Database analysis	Colorectal Laparoscopic Cancer/Benign	**203 ERP > 75y** **175 ERP 66-75y** 211 ERP ≤65y	**80 (range NR)** **69 (range NR)** 56 (range NR)	Colorectal resection Scheduled for laparoscopic approach	Emergency surgery Palliative procedure
Forsmo 2017 [18]	Norway Monocentric, academic	Secondary analysis of RCT data	Colorectal Open/Lap Cancer/Benign	**19 ERP ≥ 80y** **56 ERP 66-79y** 79 ERP ≤ 65y	**83 (80–89)** **72 (66–78)** 58 (23–65)	≥18y Colorectal surgery With or without stoma Malignant or benign	Multi-organ resection ASA 4 Emergency surgery Impaired mental capacity
Braga 2017 [19]	Peri-operative Italian Society Registry (11 hospitals)	Database analysis	Colorectal Open/Lap Cancer/Benign	**93 ERP > 80 y** **117 ERP 76-80y** **105 ERP 71-75y**	**84 (SD ± 3)** **77 (SD ± 2)** **73 (SD ± 1)**	>70y Elective colorectal surgery	/
Braga 2016 [20]	Peri-operative Italian Society Registry (11 hospitals)	Database analysis	Colorectal Open/Lap Cancer/Benign	**167 ERP ≥ 70y, ASA 1–2** **162 ERP ≥ 70y, ASA 3–4** 279 ERP <70y, ASA 1–2 98 ERP <70y, ASA 3–4	**77 (SD ± 4.6)** **78 (SD ± 5.3)** 58 (SD ±9) 63 (SD ±5.7)	Elective colorectal surgery	/
Gonzalez-Ayora 2016 [21]	Spain Multicentric, academic	Database analysis	Colorectal Open/Lap Cancer/Benign	**188 ERP ≥ 70y**	**79 (70–93)**	≥70y Colorectal surgery	Emergency surgery Palliative procedure
Pedziwiatr 2015 [22]	Poland Monocentric, academic	Database analysis	Colorectal Laparoscopic Cancer	**34 ERP ≥ 80y** 43 ERP ≤55y	**83 (IQR 82–87)** 50 (IQR 44–54)	≥80y or ≤ 55y Laparoscopic colorectal surgery Colorectal adenocarcinoma	Emergency surgery Multi-organ or transanal resection Concomitant IBD ICU stay after surgery
Kisialeuski 2015 [23]	Poland Monocentric, academic	Prospective observational cohort study	Colorectal Laparoscopic Cancer	**49 ERP > 65y** 43 ERP ≤65y	**76.3 (SD NR)** 55.8 (SD NR)	Laparoscopic colorectal surgery Colorectal cancer	Emergency surgery Multi-organ resection
Jia 2014 [24]	China Monocentric, academic	RCT	Colorectal Open Cancer	**117 ERP ≥ 70y** 116 CC ≥70y	**75.7 (SD ± 4.2)** 74.8 (SD ±4.0)	≥70y Admitted for open curative resection Colorectal carcinoma	Dementia, Parkinson, alcohol intake ≥250 g/d, long term sleeping pills or anxiolytics, anaesthesia ≤30d Intra-operative blood transfusion or ICU stay after surgery
Keller 2013 [25]	USA Monocentric, academic	Database analysis	Colonic Laparoscopic Cancer/Benign	**153 ERP ≥ 70y** 302 ERP <70y	**77.9 (SD ± 6.1)** 52.4 (SD ±13.7)	Elective laparoscopic colon resection (conversions included)	Incomplete medical or financial records
Feroci 2013 [26]	Italy Monocentric, non-academic	Database analysis	Colorectal Open/Lap Cancer/Benign	**204 ERP ≥ 75y** 402 ERP <75y	Overall: 70 (30–94)	Elective colorectal resection (multiple previous laparotomies are included) ASA grades 1 to 4	Medically unfit for surgery Cancer with distant metastasis <18y or pregnant
Baek 2013 [27]	Korea Monocentric, academic	Prospective observational cohort study	Colorectal Laparoscopic Cancer	**77 ERP ≥ 70y** 226 ERP <70y	**74.8 (SD ± 4.2)** 56.7 (SD ±8.9)	Laparoscopic or robotic surgery Colorectal cancer	Emergency surgery ASA 4 ICU stay after surgery Conversion (laparoscopic to open)
Wang 2012 [28]	China Monocentric, academic	RCT	Colorectal Laparoscopic Cancer	**40 ERP ≥ 65y** 38 CC ≥65y	**71 (65–81)** 72 (65–82)	≥ 65y Laparoscopic colorectal resection Colorectal cancer	Distant metastasis (involving pelvic, urethra of iliac vessel invasion) Poor cardiopulmonary function
Pawa 2012 [29]	UK Monocentric, academic	Database analysis	Colorectal Open/Lap Cancer/Benign	**130 ERP ≥ 80y** 558 ERP < 80y	**83 (80–95)** 66 (17–79)	Colorectal resection	None
Walter 2011 [30]	UK Monocentric, non-academic	Database analysis; retrospective control group	Colorectal Open/Lap Cancer/Benign	**68 ERP ≥ 80y** 332 ERP < 80y 200 CC	Overall: 67 (IQR 56–77) 69 (IQR 57–78)	Major colorectal resections First 400 consecutive, non-selected, patients managed within an ERP Last 200 patients pre-ERP	Emergency surgery
Kahokehr 2011 [31]	New Zealand Monocentric, academic	Prospective observational cohort study	Colonic Open/Lap Cancer/Benign	**22 ERP > 75y** 78 ERP ≤75y	Overall: 67.5 (IQR 31–92)	Elective colonic surgery within an ERP	Rectal cancer ≤15 cm from the anal verge, patients requiring a stoma or unable to participate (language, cognitive impairment, ASA ≥4)
Rumstadt 2009 [32]	Germany FTCII programme (24 hospitals)	Database analysis	Colonic Open/Lap Cancer/Benign	**207 ERP ≥ 80y** **535 ERP 70-79y**	**74.7 (70–79.9)** **83.4 (80–95.7)**	≥ 70y Elective colonic resection	Emergency surgery Perforation or abscess with septic inflammatory response syndrome
Hendry 2009 [33]	UK, Norway, Sweden, The Netherlands Multicentric, academic	Database analysis	Colorectal Open Cancer/Benign	**194 ERP ≥ 80y** 839 ERP <80	Overall: 59 (IQR 69–78)	Elective open colorectal surgery with formation of an anastomosis In case of rectal cancer: tumour in the upper 1/3 of the rectum and allows anastomosis in the middle 1/3 ASA grade 1 to 4	Total mesorectal excision
Scharfen-berg 2007 [34]	Germany Monocentric, academic	Prospective observational cohort study	Colonic Open/Lap Cancer/Benign	**74 ERP > 70y**	**74 (71–88)**	> 70y Elective colonic resection Benign or malignant disease	Not operated on electively
Senagore 2003 [35]	USA Monocentric, academic	Retrospective observational cohort study	Colonic Open/Lap Cancer/Benign	**50 ERP ≥ 70y, lap** **123 ERP ≥ 70y, open** 181 ERP <60y, lap 122 ERP <60y, open	**77.5 (SE ± 4.6)** **77.8 (SE ± 5.4)** 42.4 (SE ±12.3) 46.7 (SE ±9.8)	4 age-matched cohorts Elective segmental colectomy Laparoscopic/open when excluded for laparoscopic approach based on standardised criteria	Prior major abdominal surgery Incomplete data
Bardram 2000 [36]	Denmark Monocentric, academic	Retrospective observational cohort study	Colonic Laparoscopic Cancer/Benign	**39 ERP ≥ 70y, lap** 11 ERP ≥70y, converted	Overall: 81 (70–93)	Laparoscopic colonic resection Laparoscopic surgery 70–75y: benign disease or malignant disease with severe cardiopulmonary disease > 75y: malignant disease	Not elective Tumours in the transverse colon or rectum Patients not self-caring and not admitted directly from home

LEGEND: *RCT* randomised controlled trial, *ERP* enhanced recovery programme, *CC* conventional care, *vs* versus, *y* years old, *g* gram, *d* day, *lap* laparoscopic, *SD* standard deviation, *SE* standard error of the mean, *NR* not reported, *TNM* tumour node metastasis, *ASA* American society of anaesthesiologists physical status class, *IBD* inflammatory bowel disease, *ICU* intensive care unit, *FTCII* fast track colon II open quality assurance programme; Bold: patient group included in this review

### Risk of bias (quality) assessment

The scores on the MINORS quality and risk of bias assessment ranged from 8 to 14 out of 16 (Additional file 2). Only 8 studies had a clearly stated aim, including the population, the intervention and at least one primary outcome. Although not always divided into primary and secondary endpoints, all studies had endpoints appropriate to the aim of the study. Lower total scores were due to non-consecutive inclusion of patients, non-prospective collection of data, absence of prospective study size calculation, or unblinded assessment of the study endpoints.

### Components of the ERP

The number of ERP intervention components in the described ERP protocols varied between 7 and 16, with a median of 13 (Table 2). All programmes described early post-operative mobilisation, early post-operative oral intake, opioid sparing multimodal post-operative analgesia, early urinary catheter removal, and avoidance of nasogastric tubes as part of the intervention. Prevention of post-operative ileus (by chewing gum, laxatives, or Alvimopan), post-operative nausea and vomiting (PONV) prophylaxis or treatment, avoidance of sedative premedication, and pre-operative optimisation were not mentioned in over half of the studies. None of the ERPs mentioned peri-operative glycaemic control. Anaesthetic protocol information was often very limited. Therefore the scoring for ‘standard anaesthesia protocol’ was based on the described regional anaesthesia technique, taking into account an evolution in the recommendations over time: In the past epidural anaesthesia was recommended in all patients. In the 2012 ERAS® Society guidelines epidural anaesthesia remains the standard in open surgery, but for laparoscopic surgery spinal analgesia or PCIA are recommended as an alternative [3, 4].

**Table 2:** Reported intervention components of the ERP

20 Components (defined according to ERAS® Society guidelines 2012 [3, 4])	Zeng 2017 [16]	Pirrera 2017 [17]	Forsmo 2017 [18]	Braga 2017 [19]	Braga 2016 [20]	Gonzalez-Ayora 2016 [21]	Pedziwiatr 2015 [22]	Kisialeuski 2015 [23]	Jia 2014 [24]	Keller 2013 [25]	Feroci 2013 [26]	Baek 2013 [27]	Wang 2012 [28]	Pawa 2012 [29]	Walter 2011 [30]	Kahokehr 2011 [31]	Rumstadt 2009 [32]	Hendry 2009 [33]	Scharfenberg 2007 [34]	Senagore 2003 [35]	Bardram 2000 [36]	**Total per ERP component (median = 13.5)**
1. Pre-operative counselling	0	1	1	1	1	1	1	1	0	1	1	1	0	1	1	1	1	1	1	1	1	**18**
2. Pre-operative optimisation	0	0	0	0	0	1	0	1	0	0	1	0	0	1	0	0	0	0	0	0	0	**4**
3. Avoidance of bowel preparation in colonic surgery	1	1	0	1	1	1	1	1	0	0	1	1	0	0	1	1	1	0	0	0	0	**12**
4a. Limited pre-operative fasting time	1	1	1	0	0	0	0	0	1	0	1	1	0	0	1	1	0	1	0	0	0	**16**
4b. Carbohydrate loading	0	1	1	1	1	1	1	1	1	0	0	0	1	1	1	1	0	1	0	0	0	
5. Avoid sedative premedication	0	0	1	1	1	0	0	0	0	0	1	0	0	0	0	0	0	1	0	0	0	**5**
6. Prophylaxis against thromboembolism	0	1	1	1	1	1	1	1	0	1	1	1	0	1	0	0	0	0	0	0	0	**11**
7. Antimicrobial prophylaxis	0	1	1	1	1	1	1	1	0	1	1	1	0	1	0	0	0	0	1	0	1	**13**
8. Standard anaesthetic protocol^1^	1	0	1	1	1	0	1	0	1	1	1	0	1	0	1	1	1	1	1	0	1	**15**
9. PONV prophylaxis/treatment	0	0	0	1	1	0	1	1	0	0	1	0	0	0	0	1	1	1	0	0	0	**8**
10. Laparoscopy and modifications of surgical acces^2^	1	1	1	0	0	0	1	1	0	1	0	1	1	1	1	0	1	1	0	1	1	**14**
11. Avoidance of nasogastric tubes	1	1	1	1	1	1	1	1	1	1	1	1	1	1	1	1	1	1	1	1	1	**21**
12. Prevention intra-operative hypothermia	0	1	1	1	1	1	0	0	0	0	1	1	0	1	1	1	0	1	0	0	0	**11**
13. Peri-operative fluid management	1	1	1	1	1	1	1	1	0	0	0	1	1	1	1	1	1	1	1	0	1	**17**
14. Avoid abdominal or pelvic drains	1	1	1	1	1	1	1	1	1	0	0	0	0	1	1	1	1	0	0	0	0	**13**
15. Early removal of urinary catheters	1	1	1	1	1	1	1	1	1	1	1	1	1	1	1	1	1	1	1	1	1	**21**
16. Prevention of post-operative ileus^3^	0	1	1	0	0	0	0	1	0	0	0	0	1	0	0	0	1	1	1	0	0	**7**
17. Opioid sparing multimodal post-operative analgesia	1	1	1	1	1	1	1	1	1	1	1	1	1	1	1	1	1	1	1	1	1	**21**
18. Early oral intake	1	1	1	1	1	1	1	1	1	1	1	1	1	1	1	1	1	1	1	1	1	**21**
19. Peri-operative glycaemic control	0	0	0	0	0	0	0	0	0	0	0	0	0	0	0	0	0	0	0	0	0	**0**
20. Early mobilisation	1	1	1	1	1	1	1	1	1	1	1	1	1	1	1	1	1	1	1	1	1	**21**
**Total ERP components per article (median = 13)**	**11**	**15**	**16**	**16**	**16**	**14**	**15**	**16**	**8**	**10**	**15**	**13**	**10**	**14**	**13**	**13**	**13**	**14**	**10**	**7**	**10**	

LEGEND: 0 = not reported as a component of the ERP; 1 = reported as a component of the ERP; ERP: enhanced recovery programme; PONV: post-operative nausea and vomiting; ^1^based on the regional anaesthesia technique (0: no/inadequate information, epidural anaesthesia as a routine procedure for laparoscopic surgery in studies that started including after 2012; 1: epidural anaesthesia for all patients, except for patients undergoing laparoscopic surgery after 2012); ^2^automatically score 1 if only laparoscopic patients were included in the study; ^3^chewing gum or laxatives or Alvimopan

### Adherence to the ERP

Nine studies reported adherence rates to a minimum of 1 and a maximum of 15 ERP components [19–23, 26, 29, 32, 34] (Table 3). Adherence was the highest for pre- and intra-operative ERP components, and lower for post-operative components. ERP components with adherence rates ≥80% were: pre-admission counselling, no bowel preparation, limited pre-operative fasting, antithrombotic and antimicrobial prophylaxis, no nasogastric tube, active warming, and limited intra-operative fluids. Other ERP components with adherence rates ≥60% included carbohydrate loading, PONV prophylaxis, and opioid sparing analgesia. ‘Early intake of oral liquids’ ranged between 49 and 84% on post-operative day (POD) 0 and ‘early intake of solid foods’ ranged between 51 and 86% on POD 1. Early mobilisation ranged between 55 and 90% on POD 0. Three studies reported ‘global compliance’ and used the percentage of patients that fully adhered to a number of selected components: global compliance ranged between 56 and 85% [20–22].

**Table 3:** Reported adherence to the ERP components

	Braga 2017 [19]			Braga 2016 [20]		Gonzalez-Ayora 2016 [21]	Pedziwiatr 2015 [22]	Kisialeuski 2015 [23]	Feroci 2013 [26]	Pawa 2012 [29]	Rumstadt 2009 [32]		Scharfen-berg 2007 [34]	range
	71-75y	76-80y	> 80y	≥ 70y, ASA 1,2	≥ 70y, ASA 3,4	≥ 70y	≥ 80y	> 65y	≥75y	≥ 80y	70-79y	≥80 y	>70y	
	*n* = 105	*n* = 117	*n* = 93	*n* = 167	*n* = 162	*n* = 188	*n* = 34	*n* = 49	*n* = 204	*n* = 130	*n* = 535	*n* = 207	*n* = 74	
1.Pre-admission counselling	100	99	97	100	100		100		100					97–100
3.No bowel preparation	90	86	90	91	85		80		100	/	80	83	/	80–100
4a.Limited pre-operative fasting time	/	/	/	/	/	/	/	/	100	/	/	/	/	
4b.Carbohydrate loading	80	81	82	87	73	100	77		/		/	/	/	73–100
5.No sedative premedication	40	44	40	40	40	/	/	/	100	/	/	/	/	40–100
6.Antithrombotic prophylaxis	100	100	100	100	100		100		100		/	/	/	100
7.Antimicrobial prophylaxis	100	100	100	100	100		100		100		/	/		100
9.PONV prophylaxis	73	66	88	88	61	/	60			/	93	95	/	60–95
10.Minimal invasive surgery	/	/	/	/	/	/	/	/	/	93	39	25	/	25–93
11.No nasogastric tube	91	93	90	92	92		100		100					90–100
12.Active warming	99	97	95	100	100		/	/	100		/	/	/	95–100
13.Peri-operative fluid management							92		/					
- Intra-operative fluids (mean ± SD or median and IQR, in ml/kg/h)	9.7 (±4.1)	8.5 (±4.0)	10.3 (±5.9)	7.2 (4.8–10.1)	8.9 (6.1–12.6)									7.2–10.3
- Infusion < 3000 ml during surgery											87	81		81–87
- Stop IV fluid POD 1						73		/		24	75	62		24–75
- Stop IV fluid POD 2	74	67	60	70	67									60–74
14.No abdominal drain	30	33	44	37	31	43	80		/				/	30–80
15.Early (per protocol) UC removal	70	67	69	78	62	65	80		64	56				56–80
17.Multimodal opioid sparing analgesia														
- Non-opioid based analgesia							74				92	89		74–92
- Epidural analgesia	50	51	58	61	43	62	55				86	86		43–86
- Epidural catheter removal ≤ POD 3	36	38	47	78	75					69				36–78
18.Early oral intake														
- Oral liquids POD 0	59	59	49	56	57						75	69	84	49–84
- Oral liquids POD 1	90	92	90					87	46					46–92
- Oral liquids POD 0–1										84				
- Solid food POD 1	53	57	52	52	57		82			73	60	51	86	51–86
- Solid food POD 2	77	86	82			92			39					39–92
20.Early mobilisation														
- Out of bed POD 0						90			60		71	55		55–90
- Out of bed POD 1	93	91	89	95	86		94	55		20	69	53		20–95
Global Compliance				66^1^	56^1^	56^2^	85^3^							56–85

LEGEND: all results in %, unless otherwise specified; PONV: post-operative nausea and vomiting; POD: post-operative day; IV: intravenous; UC: urinary catheter; ml: millilitre; kg: kilogram; h: hour; n: number of patients; y: years old; ASA: American Society of Anaesthesiologists physical status classification; SD: standard deviation; IQR: inter quartile range; ^1^18, ^2^7 and ^3^13 elements used to assess global compliance; /: not applicable (the intervention component was not part of the ERP or (for component 10) the study included laparoscopic patients only)

### Outcomes of the ERP

Results from individual studies per study group are given in Table 4. The median post-operative morbidity rate during the hospital stay was 23.5%, ranging from 5 to 37.8% (9 study groups) and was 29.8% at 30 post-operative days, ranging from 18.8 to 52.6% (13 study groups). The in-hospital mortality rate was 0% in 8 study groups, and 1.2 and 1.6% in two others. The median mortality at 30 days was 1.4%, ranging from 0 to 16.2% (17 study groups).

**Table 4:** Outcomes of the ERP in older patients

	Zeng 2017 [16]	Pirrera 2017 [17]		Forsmo 2017 [18]		Braga 2017 [19]			Braga 2016 [20]	Gonzalez-Ayora 2016 [21]	Pedziwiatr 2015 [22]	Kisialeuski 2015 [23]	Jia 2014 [24]	Keller 2013 [25]	Feroci 2013 [26]	Baek 2013 [27]	Wang 2012 [28]	Pawa 2012 [29]	Walter 2011 [30]	Kahokehr 2011 [31]	Rumstadt 2009 [32]		Hendry 2009 [33]	Scharfenberg 2007 [34]	Senagore 2003 [35]	Bardram 2000 [36]
Age (years)	≥75	66–75	> 75	66–79	≥80	71–75	76–80	> 80	≥70	≥70	≥80	> 65	≥70	≥70	≥75	≥70	≥65	≥80	≥80	> 75	70–79	≥80	≥80	≥70	≥70	≥70
n of patients	94	175	203	56	19	105	117	93	329	188	34	49	117	153	204	77	40	130	68	22	535	207	194	74	173	39
MORBIDITY^1^ (in %)																										
- inH		21.1	21.2							37.8	23.5	36.7		17.0		26.0	5.0								31.2	
- 30d	24.5			41.2	52.6	21.0	18.8	30.1	29.8						37.3						23.0	38.2	33.0	21.6		20.5
MORTALITY (in %)																										
- inH						0	0	0		1.6		0		0		0				0				0	1.2	
- 30d	2.1			3.6	5.3	0	0	0	0.3					0	6.4			16.2	4	0	1.1	1.0	3.1	1.4		5.1
LOS (in days)																										
- Post-operative (mean, ±SD)		4.7 ± 4.5	4.7 ±5.1			6.2 ± 3.1	6.7 ± 3.5	7.3 ± 3.6			5.4 ±5	5.5 ± 4														
- Post-operative (median, range)	6 (4–21)			5 (2–21)	6.5 (3–50)				6 (IQR 5–8^1^ and 4–7^2^)		5 (IQR 3–7)				7 (3–43)	8 (4–27)	5.5 (IQR 5–6)		7 (IQR 6–10)		8 (2–83)	11 (1–53)		5 (3–56)		2.5 (2–90)
- total (mean, ±SD)													9.0 ±1.75	5.0 ±4.91												
- total (median, range)																12 (7–31)		8 (IQR 5–14)		6 (IQR 3–8)						
READMISSION (in %) - 30d		4.6	4.9	25.0	21.1	5.7	1.7	1.1	2.4	6.4	2.9	6.1		4.6	1.5	11.7		6.2	4		4.7	2.4		12.2	6.4	5.1
REOPERATION (in %)	5.3	1.2	3.4	14.3	10.5	5	5	4	5.2	8.5	0	4.1		1.3				8.5							0.6	7.7

LEGEND: ^1^postoperative complications (Clavien-Dindo severity grades I to IV); n: number; d: days; inH: in-hospital; ^1^result for ASA 1,2 patients; ^2^result for ASA 3,4 patients; ASA: American Society of Anaesthesiologists physical status classification; SD: standard deviation; IQR: inter quartile range

LOS was not reported in a uniform way: four studies reported mean post-operative hospital stay (median among the studies 5.5 days, ranging from 4.7 to 7.3 days). Eleven studies reported median post-operative hospital stay (median among the studies 6.0 days, ranging from 2.5 to 11 days). Two studies reported mean total hospital stay (5.0 and 9.0 days, respectively). Three studies reported the median total hospital stay (6.0, 8.0 and 12.0 days, respectively). Two studies reported LOS including readmission days [21, 33], and one study reported mean LOS for open and laparoscopic surgery separately (9.3 and 4.2 days, respectively) [35], which explains the absence of LOS-results for these studies in the table.

Among the 16 studies reporting the 30-day readmission rate, the median reported rate was 4.9%, ranging from 1.1 to 25.0% (21 study groups). Among the 12 studies reporting reoperation rate, the median reported rate was 5.0%, ranging from 0.6 to 10.5% (16 study groups). Additional file 3 summarises the attainment of ERP recovery goals. Time to readiness for discharge (TRD or achievement of all recovery goals) was reported in 3 studies with a mean of 6.4, a median of 5.0, and a median of 5.5 days, respectively [19, 20, 32].

## Discussion

This review analysed intervention components, adherence and outcomes of ERPs in 21 studies in older elective colorectal surgical patients.

The median number of ERP intervention components per study was 13 (range 7–16), compared to 10 (range 4–18) in the earlier review by Bagnall et al. (2014) [11]. When comparing the studies included in our review, those published before 2014 implemented a median of 13 (range 7–15), and those after 2014 a median of 15.5 (range 11–16) components. This demonstrates that more recent ERPs implement more ERP intervention components than older programmes do. To facilitate comparison of studies, the ERP interventions were grouped into 20 key components based on the 2012 ERAS® Society guidelines [3, 4]. This explains minor differences in the reported number of ERP components per study between this review and the review by Bagnall et al., as well as minor differences with what authors of the included studies report. Although only 4 studies were conducted after the publication of the 2012 ERAS® Society guidelines, the ERP components in these guidelines do not differ substantially from previous consensus recommendations [37, 38], nor do they differ from more recent guidelines by other societies (e.g. from the United States, France) [39, 40]. It is worth mentioning that some components remain somewhat vague in the guidelines: for example, no exact time frame is given for ‘early feeding’ and ‘early mobilisation’, nor is the kind of food and the intensity of physical exercise specified. The included studies therefore construct their own protocols, which leads to heterogeneity.

Some ERP components, e.g. pre-operative optimisation, antithrombotic and antimicrobial prophylaxis, PONV prophylaxis and treatment, were less often reported than expected. Possibly, these components are considered peri-operative routine and are therefore not systematically mentioned by the authors in the ERP protocol. Tight peri-operative glycaemic control with insulin was not reported in any of the ERPs. We hypothesize this is due to the fact that it only appeared as an ERP component in the latest^1^ guideline in 2012, with a warning for the risk of hypoglycaemia in a ward setting [3, 37, 38].

To allow for a correct interpretation of the effectiveness of the programmes, adherence to individual ERP components, i.e. which ERP components the patients actually receive (dependent on the providers) and which ones they are able to carry out or tolerate (dependent on the patients), needs to be reported as well [18, 41, 42]. Nine studies (43%) reported adherence, although the difference between interventions offered and tolerated was often unclear, and adherence was only given for a selection of ERP components. Former studies demonstrated that ERP adherence is lower in the post-operative period compared to the pre- and intra-operative period, and this seems equally true for the older patients in our review [43–45]. This should not automatically be considered as an implementation failure: declining adherence in the post-operative phase might reflect the development of complications, for which additional measures can be taken [44]. Five studies compared adherence in older versus younger patients [20, 22, 23, 26, 29], two in different older age groups [19, 32], and two studies described adherence in a single older age group [21, 34]. As the age limits of the groups varied and the ERP components assessed were too heterogeneous or limited in number, it was not possible to draw firm conclusions about adherence in relation to age. It was not possible either to draw conclusions about the factors that affect adherence among different age groups, such as location [19, 21, 26] and invasiveness [21] of the surgery, placement of a stoma [21, 26], and cancer diagnosis [19, 26]. Only a higher ASA class seems consistently associated with lower adherence [20, 26]. Possibly, ability to adhere to the programme in older patients may also be influenced by age-related factors: co-morbidity, frailty, functional impairments, cognitive impairments, ageism, or lack of personnel or materials (e.g. physiotherapists or walking aids) to take care of the more challenging older patients. Nevertheless, none of the presumed age-related factors above were studied.

A secondary objective of this review was to summarise outcomes of ERPs in older patients, without comparison to younger patients or conventional care: the two earlier reviews studied the safety and feasibility of ERPs (by analysing cohort studies comparing different age groups), and their efficacy (by analysing the only two existing RCTs of ERP versus conventional care in older patients [24, 28]). They concluded that ERPs are safe and feasible, with a comparable post-operative morbidity in the younger and older patient population in the majority of the studies, and that ERPs had significantly better outcomes than conventional care [11, 12]. In this novel review we included 8 new studies (we excluded emergency surgery, which explains why two studies from the previous reviews were not included). The outcomes in the new studies are comparable to those in the older studies. Unfortunately, differences in age ranges of recruited patients, differences in in- and exclusion criteria of the studies, differences in ERP pathways, and differences in definitions of outcomes or poorly defined ERP components and outcomes, and incomplete reporting of adherence preclude further causative analysis. Since the details of individual complications were very heterogeneously reported in studies, we decided to report overall complication rates. Only one study reported the incidence of postoperative delirium [24].

Some methodological aspects need to be taken into account while interpreting the review results. First, only six studies included older patients or subgroups of older patients prospectively [23, 24, 27, 28, 31, 34]. The other studies were secondary analyses, database analyses or retrospective studies. Second, often only subgroups of patients from the original studies were included. For this reason, it was not possible to give more details about the included older population in terms of ratios for colon versus rectal surgery, laparoscopic versus open surgery, cancer versus benign disease, or ASA class. Third, the many differences among studies precluded meta-analysis of the results. Better quality research and standardised reporting is needed to draw conclusions on the optimal composition and the outcomes of the programme in older patients. Fourth, the included studies only report the chronological and not the biological age of their patients in terms of co-morbidity, frailty, mental capacity and functional dependency. The older patients in this review may be apt to selection bias, as mainly physically and mentally fit patients tend to be recruited in studies, and there might also be a referral bias [46]. A strength of this review is the comprehensive search string with selection of papers in five different languages, without limitations on study design, and without searching for ‘older patients’. This ensures a comprehensive overview of publications from the last 17 years describing the components, the adherence and the outcomes in (subgroups of) patients aged over 65 undergoing elective colorectal surgery.

The data provided by this review will be of added value to guide clinical decision-making and patient counselling. In addition, clinicians and researchers can use this overview as a reference to evaluate their own data. For future studies, there is a need to standardise and further fine-tune definitions of ERP intervention components and outcomes, and to provide guidance for standardised reporting [42, 47]. This will facilitate comparison among studies and allow meta-analysis. Adherence for all of the ERP components should be reported in a uniform way, and should reflect which ERP components patients actually receive, as well as which interventions they are able to carry out or tolerate. Large studies should describe patient characteristics, adherence and outcomes for their older patient groups in detail, to enable secondary analysis of the older patient population. Future studies and (inter)national audit initiatives will have to be specifically designed to study older patients in relation to their frailty profile, preferably incorporating a comprehensive geriatric assessment for the evaluation of the older individual’s functional, cognitive and psychosocial status, comorbidities and polypharmacy [10, 48, 49]. Based on these findings, it will be possible to thoroughly describe the older population studied. This will allow to determine whether frail older patients can follow a standard ERP or whether it should be tailored to this specific population, and to interpret outcomes in relation to older patients’ profiles.

## Conclusions

The ERP components applied in older patient populations were similar to those described in the ERP consensus guidelines, and the number of intervention components in the ERPs increased over time. Nevertheless, outcomes in earlier and later studies remained comparable. Although important to interpret outcomes, adherence rates were rarely reported. The pre- and intra-operative adherence (more dependent on the providers) was higher than the post-operative adherence (more influenced by patient-related factors). Future studies should explore adherence and age-related factors that could influence adherence, such as frailty profile.

## Additional files

Additional file 1: Search strategy for PUBMED, CINAHL and EMBASE. (DOCX 13 kb)

Additional file 2: MINORS quality assessment. (DOCX 43 kb)

Additional file 3: Attainment of ERP recovery goals. (DOCX 32 kb)

## Abbreviations

ASA: American society of anaesthesiologists physical status class
ERAS: Enhanced recovery after surgery
ERP: Enhanced recovery programme
GC: Global compliance
LOS: Length of stay
MINORS: Methodological index for non-randomised studies
PCIA: Patient controlled intravenous analgesia
POD: Post-operative day
PONV: Post-operative nausea and vomiting
RCT: Randomised controlled trial
TRD: Time to readiness for discharge

## Footnotes

1
*An update of the 2012 ERAS® Society guideline is expected by the end of 2018.*
