# Supplementary material for: A systematic review of the intervention components, adherence and outcomes of enhanced recovery programmes in older patients undergoing elective colorectal surgery
Source: BMC Geriatr. 2019 Jun 6;19:157. doi: 10.1186/s12877-019-1158-3 (PMC6555702; doi:10.1186/s12877-019-1158-3)
Supplement: Supplementary file 1 — Search strategy for PUBMED, CINAHL and EMBASE. (DOCX 13 kb) [file 12877_2019_1158_MOESM1_ESM.docx]

Additional file 1: Search strategy for PUBMED, CINAHL and EMBASE

| Pubmed | (((“colorectal surgery”[mesh]) OR ((colorectal[tiab] OR colon[tiab] OR colonic[tiab] OR colectomy[tiab] OR rectum[tiab] OR rectal[tiab] OR pelvic[tiab]) AND (surgery[tiab] OR surgical[tiab] OR operation[tiab] OR operative[tiab] OR resection[tiab]))) AND (ERAS[tiab] OR “enhanced recovery” [tiab] OR “accelerated recovery” [tiab] OR “expedited recovery” [tiab] OR “fast track” [tiab] OR multimodal[tiab] OR multi-modal[tiab])) |
| --- | --- |
| Embase | ('colon surgery'/exp OR 'colorectal surgery'/exp OR 'rectum surgery'/exp OR 'colon resection'/exp OR 'rectum resection'/exp OR ( (colorectal:ti,ab OR colon:ti,ab OR colonic:ti,ab OR colectomy:ti,ab OR rectum:ti,ab OR rectal:ti,ab OR pelvic:ti,ab) AND (surgery:ti,ab OR surgical:ti,ab OR operation:ti,ab OR operative:ti,ab OR resection:ti,ab))) AND (eras:ti,ab OR 'enhanced recovery':ti,ab OR 'accelerated recovery':ti,ab OR 'expedited recovery':ti,ab OR 'fast track':ti,ab OR multimodal:ti,ab OR 'multi modal':ti,ab) |
| Cinahl | (((MH "Colectomy+") OR (((TI colorectal) OR (AB colorectal) OR (TI colon) OR (AB colon) OR (TI colonic) OR (AB colonic) OR (TI colectomy) OR (AB colectomy) OR (TI rectum) OR (AB rectum) OR (TI rectal) OR (AB rectal) OR (TI pelvic) OR (AB pelvic)) AND ((TI surgery) OR (AB surgery) OR (TI surgical) OR (AB surgical) OR (TI operation) OR (AB operation) OR (TI operative) OR (AB operative) OR (TI resection) OR (AB resection)))) ) AND ( (TI ERAS) OR (AB ERAS) OR (TI “enhanced recovery”) OR (AB “enhanced recovery”) OR (TI “accelerated recovery”) OR (AB “accelerated recovery”) OR (TI “expedited recovery”) OR (AB “expedited recovery”) OR (TI “fast track”) OR (AB “fast track”) OR (TI multimodal) OR (AB multimodal) OR (TI multi-modal) OR (AB multi-modal)) |
