# Supplementary material for: A systematic review of the intervention components, adherence and outcomes of enhanced recovery programmes in older patients undergoing elective colorectal surgery
Source: BMC Geriatr. 2019 Jun 6;19:157. doi: 10.1186/s12877-019-1158-3 (PMC6555702; doi:10.1186/s12877-019-1158-3)
Supplement: Supplementary file 2 — MINORS quality assessment. (DOCX 43 kb) [file 12877_2019_1158_MOESM2_ESM.docx]

Additional file 2: MINORS quality assessment [15]

| **Study** | Clearly stated aim | Inclusion of consecutive patients | Prospective collection of data | Endpoints appropriate to the aim of the study | Unbiased assessment of the study endpoint | Follow-up period appropriate to the aim of the study | Loss to follow up < 5% | Prospective calculation of the study size | **Total** |
| --- | --- | --- | --- | --- | --- | --- | --- | --- | --- |
| Zeng 2017 [16] | 2 | 2 | 1 | 2 | 0 | 2 | 2 | 0 | **11** |
| Pirrera 2017 [17] | 1 | 2 | 1 | 2 | 0 | 2 | 2 | 0 | **10** |
| Forsmo 2017 [18] | 1 | 0 | 2 | 2 | 1 | 2 | 2 | 0 | **10** |
| Braga 2017 [19] | 1 | 0 | 1 | 2 | 0 | 2 | 2 | 0 | **8** |
| Braga 2016 [20] | 1 | 0 | 1 | 2 | 0 | 2 | 2 | 0 | **8** |
| Gonzalez-Ayora 2016 [21] | 2 | 2 | 1 | 2 | 0 | 2 | 2 | 0 | **11** |
| Pedziwiatr 2015 [22] | 1 | 2 | 1 | 2 | 0 | 2 | 2 | 0 | **10** |
| Kisialeuski 2015 [23] | 1 | 0 | 2 | 2 | 0 | 2 | 2 | 0 | **9** |
| Jia 2014 [24] | 2 | 0 | 2 | 2 | 0 | 2 | 2 | 0 | **10** |
| Keller 2013 [25] | 2 | 0 | 1 | 2 | 0 | 2 | 2 | 0 | **9** |
| Feroci 2013 [26] | 1 | 2 | 1 | 2 | 1 | 2 | 2 | 0 | **11** |
| Baek 2013 [27] | 2 | 0 | 2 | 2 | 0 | 2 | 2 | 0 | **10** |
| Wang 2012 [28] | 2 | 2 | 2 | 2 | 2 | 2 | 2 | 0 | **14** |
| Pawa 2012 [29] | 1 | 0 | 1 | 2 | 0 | 2 | 2 | 0 | **8** |
| Walter 2011 [30] | 1 | 2 | 1 | 2 | 0 | 2 | 0 | 0 | **8** |
| Kahokehr 2011 [31] | 1 | 2 | 2 | 2 | 0 | 2 | 2 | 0 | **11** |
| Rumstadt 2009 [32] | 1 | 0 | 1 | 2 | 1 | 2 | 2 | 0 | **9** |
| Hendry 2009 [33] | 2 | 2 | 1 | 2 | 0 | 2 | 2 | 1 | **12** |
| Scharfenberg 2007 [34] | 2 | 2 | 2 | 2 | 0 | 2 | 2 | 0 | **12** |
| Senagore 2003 [35] | 1 | 2 | 1 | 2 | 0 | 2 | 2 | 0 | **10** |
| Bardram 2000 [36] | 1 | 2 | 1 | 2 | 0 | 2 | 2 | 0 | **10** |
| LEGEND: 0 = not reported; 1 = reported but inadequate; 2 = reported and adequate | | | | | | | | | |
