# Supplementary material for: A systematic review of the intervention components, adherence and outcomes of enhanced recovery programmes in older patients undergoing elective colorectal surgery
Source: BMC Geriatr. 2019 Jun 6;19:157. doi: 10.1186/s12877-019-1158-3 (PMC6555702; doi:10.1186/s12877-019-1158-3)
Supplement: Supplementary file 3 — Attainment of ERP recovery goals. (DOCX 32 kb) [file 12877_2019_1158_MOESM3_ESM.docx]

Additional file 3: Attainment of ERP recovery goals

| **IN-HOSPITAL RECOVERY**  median (range) or mean (±SD)  in days (or hours*) | Zeng 2017 [16] | Forsmo 2017 [18] | | Braga 2017 [19] | | | Braga 2016 [20] | | Pedziwiatr 2015 [22] | Kisialeuski 2015 [23] | Jia 2014 [24] | Baek 2013 [27] | Wang 2012 [28] | Rumstadt 2009 [32] | | Hendry 2009 [33] | Scharfenberg 2007 [34] |
| --- | --- | --- | --- | --- | --- | --- | --- | --- | --- | --- | --- | --- | --- | --- | --- | --- | --- |
| Age (years) | ≥75 | 66-79 | ≥80 | 71-75 | 76-80 | >80 | ≥70 | ≥70 | ≥80 | >65 | ≥70 | ≥70 | ≥65 | 70-79 | ≥80 | ≥80 | ≥70 |
| n of patients | 94 | 56 | 19 | 105 | 117 | 93 | 167  ASA 1,2 | 162  ASA 3,4 | 34 | 49 | 117 | 77 | 40 | 535 | 207 | 194 | 74 |
| Laparoscopic / Open | L | L&O | | L&O | | | L&O | | L | L | O | L | L | L&O | | O | L&O |
| **Time until ambulation** | 1  (1-4) |  |  | 3.0 (±2.2) | 3.6 (±3.0) | 3.5 (±1.6) | 3  (IQR 2-4) | 3  (IQR 2-4) |  |  |  |  | 12*  (10-14) |  |  | 4  (2-6) |  |
| **Time to first flatus** | 2  (1-6) |  |  | 2.7 (±1.3) | 3.4 (±1.9) | 3.3  (± 1.6) | 3  (IQR 2-4) | 3  (IQR 2-4) | 1.8 (±1.2) |  | 48.5* (±9.59) | 2  (0-7) | 31*  (26-40) |  |  |  |  |
| **Time to first stool** | 3  (1-7) |  |  |  |  |  |  |  |  | 2.59 (±1.75) |  | 3  (0-6) | 55*  (48-63) |  |  |  | 2  (0-6) |
| **Time to oral intake** | 1  (1-4) | 2  (0-8) | 1  (0-9) | 2.8 (±2.3) | 3.1 (±2.7) | 3.0 (±2.0) | 3  (IQR 2-4) | 2  (IQR 2-4) |  |  |  | 2  (1-12) | 12*  (11-16) |  |  |  |  |
| **Pain control with oral drugs** |  |  |  | 3.0 (±1.3) | 3.2  (± 1.8) | 3.4 (±1.3) | 3  (IQR 2-4) | 3  (IQR 2-4) |  |  |  |  |  |  |  |  |  |
| **No morbidity evidence** |  |  |  | 5.5 (±2.6) | 5.9 (±3.1) | 6.2 (±3.0 | 5  (IQR 4-6) | 4  (IQR 3-6) |  |  |  |  |  |  |  |  |  |
| **TRD** |  |  |  | 5.7 (±2.8) | 6.4  (± 3.4) | 7.0  (± 3.4) | 5  (IQR 4-6) | 5  (IQR 3-6) |  |  |  |  |  | 5  (2-83) | 6  (3-32) |  |  |
| LEGEND: n: number; L: laparoscopic surgery; O: open surgery; ASA: American Society of Anaesthesiologists physical status classification; SD: standard deviation; IQR: inter quartile range; TRD: time to readiness for discharge (time to reach all recovery goals) | | | | | | | | | | | | | | | | | |
